# Assessment of the Connection Between Maxillary Sinus Volume, Number of Surgical Interventions, and Craniofacial Development in Patients with Unilateral Cleft Lip and Palate: A Retrospective Cross-Sectional Study

**DOI:** 10.3390/jcm14238468

**Published:** 2025-11-28

**Authors:** Aleksandra Kołodziejska, Wojciech Nazar, Jolanta Kalinowska, Bogna Racka-Pilszak, Anna Wojtaszek-Słomińska

**Affiliations:** 1 Department of Conservative Dentistry, Faculty of Medicine, Medical University of Gdańsk, 80-210 Gdańsk, Poland; 2 Laboratory of Experimental and Translational Allergology, Department of Allergology, Faculty of Medicine, Medical University of Gdańsk, Poland, Smoluchowskiego 17, 80-214 Gdańsk, Poland; 3 Medical University of Gdańsk, 80-210 Gdańsk, Poland

**Keywords:** maxillary sinus, cleft lip, cleft palate, surgery, orthodontics

## Abstract

**Objectives**: The aim of this retrospective cross-sectional study was to compare maxillary sinus volume, the number of surgical interventions, and craniofacial development in patients with unilateral cleft lip and palate (UCLP). The study also sought to clarify the surgical effect on craniofacial growth. **Materials and Methods**: The study examined 30 patients. Computed tomography scans and lateral cephalograms were collected. The volumes of the right and left maxillary sinuses were measured using ITK-SNAP software version 4.2.2 with a semiautomatic segmentation method. Cephalometric analysis was performed, and the number of primary and secondary interventions was noted. Data were statistically analyzed, with the significance set at *p* < 0.05. **Results**: Statistical analysis revealed no significant association (*p* > 0.05) between the number of primary or secondary surgical procedures and their influence on maxillary sinus volume. A significant relationship was observed between the number of primary surgeries and the ANB angle (*p* = 0.034) and SNB angle (*p* = 0.005). Statistical significance was also found between the number of secondary surgeries and the SNA angle (*p* = 0.03) and ANB angle (*p* = 0.03). **Conclusions**: The results show that the number of primary and secondary surgeries does not affect maxillary sinus volume in growing UCLP patients. However, the number of primary surgeries may have some influence on subsequent profile development. The number of secondary surgeries appears to influence maxilla growth and, consequently, the maxilla–mandible relationship (ANB and WITS appraisal).

## 1. Introduction

Cleft lip and palate are among the most prevalent craniofacial deformities in humans. The occurrence of cleft palate combined with cleft lip ranges from 5.95 to 8.91 per 10,000 live births [1]. Clefts develop in between the 7th and 12th week of gestation, influencing many functions of the newborn’s craniofacial region [2]. One of the structures within this region is the maxillary sinus. Around the 10th week of gestation, the maxillary sinus begins developing from the mucosa of the primitive ethmoidal infundibulum [3]. The greatest growth intensity is seen during the first three years of life, and the continued development of the maxillary sinus corresponds with tooth eruption. Growth ceases around age 15, when the upper second permanent molars erupt [4].

The nasal cavities and sinuses, including the maxillary sinuses, act as a primary defense mechanism of the respiratory system, protecting it against inhaled pollutants, allergens, and pathogens. The mucus secretion and rich blood supply allow the nose to heat and humidify inhaled air, which is essential for the function and health of the lower respiratory tract. The maxillary sinuses also reduce skull bone mass while maintaining facial contour, improving vocal resonance, and acting as a crumple zone during midface trauma [5,6]. In cleft patients, the maxillary sinuses are prone to sinusitis, phonation, and hearing disorders [7].

Closure of the cleft is a process that restores anatomical structure and function. Surgical closure is typically performed in the first year of life [8]. Multiple surgical protocols exist, but they are mainly divided into primary and secondary surgical procedures. Primary procedures involve the initial closure of the cleft, while secondary interventions are performed later and may include secondary alveolar bone grafting, lip repair revision, and closures of oronasal fistulas [8]. Minimizing the number of surgical interventions is essential, as postoperative scarring may inhibit future craniofacial growth [9,10].

Cone-beam computed tomography (CBCT) enables the evaluation of anatomical structures hidden inside the bony contour of the maxilla. It has proven to be an accurate method for assessing distances and parameters of the bony structures [11]. Advances in software have enabled volumetric measurements of organs, cavities, and other anatomical areas, with manual, semi-automated, and fully automated methods now available [12,13].

Interest in evaluating maxillary sinus volume in patients with cleft lip and palate has been steadily rising. Studies have compared maxillary sinus volume in cleft and non-cleft groups [14,15]. However, there is a paucity of data examining the influence of surgical interventions and treatment history on subsequent maxillary sinus and craniofacial growth. To the best of the authors’ knowledge, no study has assessed the correlation between the maxillary sinus volume—measured using a three-dimensional (3D) segmentation method—the number of surgical interventions, and the further growth of the craniofacial complex across different diagnostic modalities. The null hypothesis was that the number of primary and secondary interventions does not influence subsequent craniofacial growth or maxillary sinus volume. The aim of this retrospective study was to compare maxillary sinus volume, the number of surgical interventions, and craniofacial development in patients with UCLP.

This study was conducted in accordance with the guidelines of the Declaration of Helsinki and was approved by the Ethics Committee of the Medical University of Gdansk, Poland (Reference no. KB/163/2025, 4 April 2025). Informed consent was obtained from all subjects. Reporting followed the Strengthening the Reporting of Observational Studies in Epidemiology (STROBE) guidelines [16].

## 2. Materials and Methods

### 2.1. Study Group

In this retrospective, cross-sectional, single-center study, the radiographic database of the Orthodontic Department was searched from 2017 to 2025. No prospective cases were included. Thirty patients with UCLP were enrolled. All patients were between 9 and 20 years of age and presented UCLP: 24 with left-side UCLP and 6 with right-side UCLP. The study consisted of 16 males and 14 females. Patients were treated using 3 different surgical protocols. In 5 patients, the cleft was closed completely during a single surgery. Twenty-three patients underwent 2 surgeries to close the cleft, and the remaining two patients had 3 primary surgeries. When secondary interventions were performed, their number was noted.

### 2.2. Inclusion and Exclusion Criteria

Patients diagnosed with UCLP who had undergone at least one surgical intervention and had a complete medical record (a medical chart including surgical history, a CBCT scan covering the region of interest, and a lateral cephalogram obtained within ±2 months of the CBCT acquisition) were included in the study. Patients with other craniofacial deformities were excluded. Patients who did not meet these criteria were also excluded.

### 2.3. Data Collection

Demographic data, along with information on the number and of primary and secondary surgical interventions, cephalometric analyses, and CBCT scans, were extracted from the patients’ medical records. Patient qualification was performed from 7 April 2025 to 7 May 2025. Measurements were performed from 7 May to 1 July.

### 2.4. Radiographic Data Acquisition

The radiographic data were obtained using a Carestream CS9300C scanner (Carestream Dental LLC, Atlanta, GA, USA). The CBCT parameters were 90 kVp, 4.0 mAs, an 8.0 s scanning time, an axial layer thickness of 0.18 mm, and a voxel size of 0.30 mm^3^. Lateral cephalograms were acquired using the same scanner with the following imaging parameters: 74 kVp, 12 mAs, a 0.63 s scanning time, an axial layer thickness of 0.5 mm, and a 18 × 24 cm field of view. Following the ALARA rule, no radiographs were taken specifically for this study; researchers only used data obtained during treatment. 

Cephalometric analyses according to Segner and Hasund were performed using DDP-Otho software version 3.6.0 (Częstochowa, Poland).

**Figure 1:**
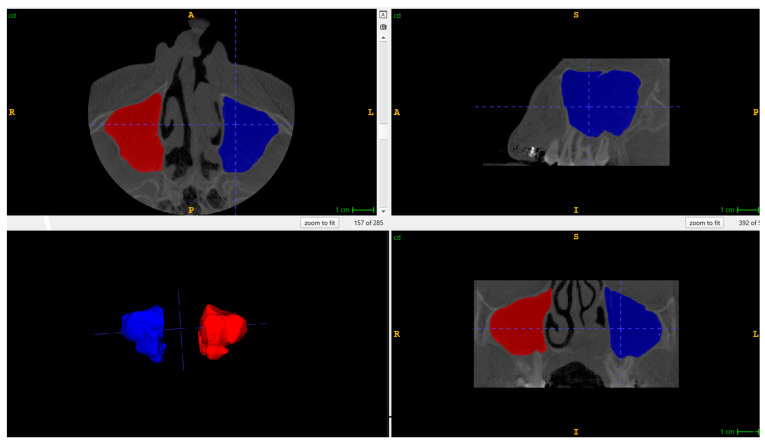
Coronal, axial, and sagittal maxillary sinus volume segmentation with 3D-generated module. Segmentation and volume calculation were performed using ITK-SNAP imaging software, version 4.2.2 (Philadelphia, PA, USA).

The CBCT data were stored as Digital Imaging and Communications in Medicine (DICOM) files. The department has used CBCT scans since 2017. The volumes of the maxillary left and right sinuses were measured and analyzed using the ITK-SNAP version 4.2.2 (Philadelphia, PA, USA) 3D imaging software package. A semiautomatic segmentation method was used to separate the maxillary sinus regions and calculate their volumes. Nearby tissues of the paranasal sinuses were removed using the Active Contour (Snake) Segmentation Mode, and pre-segmentation was performed with thresholding. The software generated 3D representations of the maxillary sinuses and calculated their volumes in cubic millimeters (Figure 1). All measurements were performed twice within a two-week interval by the first author, and repeated a month later by the third author. Both researchers were trained orthodontists with experience in CLP cases. The values represent the arithmetic means. 

### 2.5. Statistical Analysis

All statistical procedures were performed using the Statistical Package for the Social Sciences, version 30.0.0 (SPSS Inc., Chicago, IL, USA), Statistica 10 (StatSoft, Kraków, Poland), and Python 3.10, utilizing the Pandas library (v2.1.3) and Scikit-learn (v1.2.1). A two-tailed *p*-value below 0.05 was considered statistically significant. The Shapiro–Wilk test was employed to assess normality. Depending on whether data followed a normal distribution, comparisons between groups were performed using either parametric tests (such as Student’s *t*-test or ANOVA) or non-parametric alternatives (including the Mann–Whitney U test or Kruskal–Wallis test). Continuous variables were expressed either as medians with 95% confidence intervals or as means with standard deviations, depending on their distribution.

## 3. Results

This study evaluated 30 patients with UCLP (14 females and 16 males). The average age of the study group was 12 years (±5.87).

Table 1 presents a general analysis of sex, age, maxillary sinus volume, and cleft side distribution in the study group. No significant differences (*p* > 0.05) were observed between sex and age, as assessed using a paired sample *t*-test.

According to Table 1, the statistical analysis showed no statistical significance (*p* > 0.05) between the right or left maxillary sinuses in either group, showing that the cleft side did not affect the maxillary sinus volume. Therefore, a dataset of 60 sinuses was created to strengthen the value of subsequent statistical tests.

Table 2 presents maxillary sinus volume in relation to the number of primary interventions. Patients who underwent three surgical interventions for cleft closure exhibited greater maxillary sinus volumes (RMSV: 13,850.0 mm^3^ [95% CI: −53,696.2 to 81,396.2]; LMSV: 12,273.0 mm^3^ [95% CI: −16,722.6 to 41,268.6]) compared with those who underwent one (RMSV: 10,422.8 mm^3^ [95% CI: 6280.8 to 14,564.8]; LMSV: 10,041.7 mm^3^ [95% CI: 6316.5 to 13,766.8]) or two (RMSV: 12,589.8 mm^3^ [95% CI: 10,361.4 to 14,818.2]; LMSV: 12,638.0 mm^3^ [95% CI: 10,753.4 to 14,522.6]) surgeries. Patients who underwent a single surgery exhibited the smallest maxillary sinus volume compared with the other groups (RMSV: 10,422.8 mm^3^ [95% CI: 6280.8 to 14,564.8]; LMSV: 10,041.7 mm^3^ [95% CI: 6316.5 to 13,766.8]). However, these differences were not statistically significant.

As shown in Table 3, patients who did not undergo any secondary corrective surgical intervention showed a statistically insignificant difference in maxillary sinus volumes (RMSV: 12,802.2 mm^3^ [95% CI: 10,079.9–15,524.51]; LMSV: 12,758.2 mm^3^ [95% CI: 10,145.4–15,371.0]) compared with patients who underwent secondary corrective surgeries.

The relationship between the number of primary surgical corrections and the cephalometric measurements is presented in Table 4. Statistical significance was observed between the number of primary interventions and the SNB (*p* = 0.005) and ANB (*p* = 0.034) values. Moreover, patients who underwent three surgeries for cleft closure presented lower SNA values (SNA: 74.5 [95% CI: 67.5 to 81.6]) than those who underwent one (SNA: 75.3 [95% CI: 73.8 to 76.7]) or two (SNA: 76.6 [95% CI:75.1–78.2]) surgeries. Regardless of the number of interventions, patients exhibited lower SNA values compared with general norms, indicating a retrognathic maxilla. Increasing the number of interventions was associated with greater anterior rotation of the maxilla, and a reduction in anterior facial height; however, these values were not statistically significant.

The analysis of the correlation between the number of surgical corrections and the cephalometric measurements revealed statistically significant findings (Table 5). Statistical significance was observed between the number of secondary interventions and SNA (*p* = 0.03) and ANB (*p* = 0.03) values. The more secondary interventions were performed, the lower the SNA values were. 

## 4. Discussion

According to our study, the results show that the number of primary and secondary surgeries does not influence maxillary sinus volume or maxillary sinus measurements in growing cleft patients. The number of primary surgeries may influence the further development of the patient’s profile. On the other hand, the number of secondary surgeries affects maxillary growth and its relation to the mandible (ANB and WITS appraisal values).

To evaluate the influence of the cleft on maxillary sinus volume, the cleft and non-cleft sides of UCLP patients were compared. The study revealed no statistical difference in maxillary sinus volume regardless of the cleft side; the right and left sinuses were comparable. This finding corresponds with the results reported by Lopes de Rezende Barbosa et al. and Rodrigues et al. [14,15]. However, other studies have reported opposite results, showing that the maxillary sinus on the cleft side presents a smaller volume than the sinus on the non-cleft side [17,18].

The influence of the number of surgical corrections on maxillary sinus volume was also assessed. The number of primary and secondary procedures was evaluated separately. The results showed no statistically significant differences between the number of surgical procedures and their influence on maxillary sinus volume in the research group. No previous research was identified that evaluated maxillary sinus volume in correlation with the number of surgical interventions.

Studies assessing maxillary sinus volume in different craniofacial patterns in non-cleft groups differ in their results. According to Shrestha et al., class II patients present the largest maxillary sinus volume [19]; other studies do not support this finding [20,21].

A possible explanation relates to research involving maxillary bone and maxillary sinus development in growing patients. Jiang et al. investigated anatomical features of the maxilla in UCLP patients with maxillary retrusion and compared them with non-cleft class I patients. The authors reported significant differences in SNA angle values in both groups. They also evaluated the maxillary body volume and maxillary sinus volume in the right and left sinuses. Their findings show that maxillary body volume was significantly smaller in the cleft group, but maxillary sinus volume did not differ significantly between groups [22]. However, they did not report the techniques or protocols used for surgical closure of the cleft, rendering it uncertain how maxillary position and body volume influence sinus development in cleft patients. Because of the small sample size in our study, no analysis of surgical techniques and protocols was performed. 

In our research, the number of primary surgeries was not significantly associated with SNA values. However, the SNA values presented were lower than class I values, indicating a retruded maxilla. Research shows that UCLP patients who underwent surgical correction of the cleft area—regardless of the surgical protocol—showed lower SNA and ANB values, reflecting a retruded profile and tendency toward class III malocclusion compared with unoperated cleft groups. Unoperated cleft patients present higher SNA and ANB values, similar to non-cleft class I patients [9,10]. In our study, statistical significance was found for SNB and ANB values in relation to the number of primary surgeries. Lin et al. also reported maxillary growth restriction with compensatory vertical mandibular growth and clockwise rotation of the maxillofacial complex [23].

In our study, a significant correlation was found between the number of secondary surgical interventions and SNA, ANB, and WITS appraisal values. Patients who underwent more surgical interventions exhibited a class III skeletal pattern. The more surgical interventions patients underwent, the higher were the chances of presenting maxillary hypoplasia. Most secondary interventions involved secondary alveolar bone grafting, lip correction, and the closure of the residual oronasal fistulae. All of these procedures are performed in the anterior region of the maxilla. The resulting scar tissue after the procedure may inhibit further anterior maxillary growth. Kamata et al. assessed the influence of corrective surgeries on maxillary growth and concluded that, when a patient underwent more than two secondary surgeries, the likelihood of requiring further orthognathic treatment increased [24]. The influence of second alveolar bone grafting on maxillary growth is also perceived as inhibitory, according to the systematic review and the meta-analysis by Kannan [25]. On the other hand, the secondary surgeries did not affect the maxillary sinus, as they were performed in the anterior region of the face, generally involving soft tissues, with the exception of second alveolar bone grafting.

## 5. Limitations and Future Directions

A high risk of bias in the findings is noted by the authors due to the limited number of patients and age variability within the study group. A primary limitation of this retrospective study is the reliance on data from patient records, which resulted in small, heterogeneous patient groups and an inability to account for several potential confounders. Crucially, the procedures were not conducted under a standardized surgical protocol. Instead, each patient received care tailored to their individual presentation, resulting in significant variability in surgical techniques, timing of interventions, and specific aspects of postoperative care. Because of these non-standardized variables, which were beyond the control of this study design, we could not reliably stratify or adjust for their independent influence on the outcomes. Therefore, while our findings highlight important results, the effect of any single surgical variable cannot be definitively isolated. It is important to note the limitations imposed by the cross-sectional nature of our study. While we successfully identified significant relationships, this design fundamentally precludes any definitive conclusion regarding causation. Some intervention groups included only a very small number of patients, and these limited sample sizes make it challenging to draw reliable statistical conclusions. A more even distribution of patients across groups is necessary. Moreover, we cannot determine whether the factors observed directly cause the outcomes, whether the relationships are influenced by unmeasured confounding variables, or whether a different direction of association exists. Our results should therefore be interpreted as hypothesis-generating, indicating strong correlative relationships that warrant further investigation through prospective, controlled, and longitudinal studies to establish true cause-and-effect mechanisms. There was no control group included in this study. Further research with larger, more homogenous cohorts is needed.

## 6. Conclusions

According to our study, the results show that the number of primary and secondary surgeries does not influence maxillary sinus volume in growing UCLP patients. Moreover, the side of the cleft does not influence the maxillary sinus volume. The maxillary sinuses seem to develop independently. Although the number of primary surgeries may show only a minimal influence on the subsequent development of the patient’s facial profile, their overall impact appears to be limited. In contrast, the number of secondary surgeries demonstrates a more substantial effect. Our results suggest that secondary surgical interventions may influence maxillary growth and the relationship between the maxilla and mandible, as reflected in changes in ANB angle and WITS appraisal values. UCLP patients present a retruded profile and maxillary deficiency. These observations highlight the importance of carefully evaluating the necessity and timing of secondary procedures to minimize their potential impact on craniofacial development.

## Figures and Tables

**Table 1:** Descriptive statistics of the study group.

	Right UCLP	Left UCLP	*p*-Value
**Sex**			
**Female**	2	12	0.78
**Male**	4	12	0.78
**Age**	15.8 (10.3–21.3)	12.8 (11.3–14.2)	0.12
**RMSV [mm^3^]**	13,937.0 (8367.5–19,506.5)	11,816.2 (9789.0–13,843.5)	0.35
**LMSV [mm^3^]**	12,292.0 (7909.8–16,674.2)	12,045.0 (10,290.3–13,799.7)	0.89

Categorical variables were analyzed using counts and percentages. Continuous variables were summarized using medians and corresponding 95% CIs. Paired samples *t*-tests were performed with a significance level of <0.05. RMSV—right maxillary sinus volume; LMSV—left maxillary sinus volume; right UCLP—cleft present on the patient’s right side; left UCLP—cleft present on the patient’s left side.

**Table 2:** Maxillary sinus volume in relation to the number of primary interventions.

No. of Primary Interventions	1	2	3	*p*-Value
**No. of patients**	5	23	2	
**RMSV [mm^3^]**	10,422.8 (6280.8–14,564.8)	12,589.8 (10,361.4–14,818.2)	13,850.0 (53,696.184–81,396.2)	0.58
**LMSV [mm^3^]**	10,041.7 (6316.5–13,766.8)	12,638.0 (10,753.4–14,522.6)	12,273.0 (16,722.559–41,268.6)	0.40

Categorical variables were analyzed using counts and percentages. Continuous variables were summarized using medians and corresponding 95% CIs. Paired samples *t*-tests were performed with a significance level of <0.05. RMSV—right maxillary sinus volume; LMSV—left maxillary sinus volume.

**Table 3:** Maxillary sinus volume in relation to the number of secondary interventions.

No. of Secondary Interventions	0	1	2	3	6	7	*p*-Value
**No. of Patients**	7	15	5	1	1	1	
**RMSV**	12,802.2 (10,079.9–15,524.5)	10,839.0 (6596.9–15,081.1)	11,635.0 (5260.8–18,009.2)	5925.0	16,040.0	19,166.0	0.42
**LMSV**	12,758.2 (10,145.4–15,371.0)	11,079.7 (8920.1–13,239.4)	11,732.2 (6434.2–17,030.2)	5825.0	14,860.0	14,555.0	0.58

Categorical variables were analyzed using counts and percentages. Continuous variables were summarized using medians and corresponding 95% CIs. Paired samples *t*-tests were performed with a significance level of <0.05. RMSV—right maxillary sinus volume; LMSV—left maxillary sinus volume.

**Table 4:** Number of primary interventions in relation to cephalometric analysis.

No. of Primary Interventions	1	2	3	*p*-Value
**SNA** (81.00 ± 3.00)	75.3 (73.8–76.7)	76.6 (75.1–78.2)	74.5 (67.5–81.6)	0.58
**SNB** (78.00 ± 3.00)	72.6 (71.4–73.9)	75.9 (74.9–77.0)	74.0 (73.2–74.8)	0.005 *
**ANB** (3.00 ± 2.00)	1.8 (0.394–3.2)	0.646 (−0.296–1.6)	3.4 (2.4–4.4)	0.034 *
**WITS** (0.00 ± 2.00)	−0.523 (−2.849–1.8)	−1.358 (−2.562–−0.155)	1.8 (−2.15–5.8)	0.245
**SN/NL** (8.00 ± 3.00)	14.0 (10.4–17.6)	10.6 (9.5–11.8)	10.2 (7.2–13.1)	0.12

Categorical variables were analyzed using counts and percentages. Continuous variables were summarized using medians and corresponding 95% CIs. SNA—angle between sella, nasion, and point A; SNB—angle between sella, nasion, and point B; ANB—angle formed by point A, nasion, and point B; WITS appraisal—distance between perpendicular lines drawn from point A (maxilla) and point B (mandible) to the occlusal plane; SN/NL—angle between sella–nasion line and nasal line (spina nasalis anterior–pterygomaxillare). * Paired samples *t*-tests were performed with a significance level of <0.05.

**Table 5:** Secondary interventions in relation to cephalometric analysis.

No. of Secondary Interventions	0	1	2	3	6	7	*p*-Value
**SNA** (81.00 ± 3.00)	76.2 (74.4–78.0)	75.5 (73.5–77.4)	77.3 (75.9–78.7)	86.8	70.9	70.7	0.03 *
**SNB** (78.00 ± 3.00)	75.0 (73.6–76.4)	75.6 (73.7–77.5)	75.0 (73.8–76.2)	79.7	71.5	73.5	0.16
**ANB** (3.00 ± 2.00)	0.78 (−0.273–1.8)	−0.106 (−1.79–1.6)	2.3 (0.76–3.8)	7.1	−0.56	2.9	0.03 *
**WITS** (0.00 ± 2.00)	−1.456 (−2.93–0.02)	−1.457 (−3.64–0.72)	0.726 (−1.90–3.4)	4.1	−4.78	−0.3	0.11
**SN/NL** (8.00 ± 3.00)	12.2 (10.4–13.9)	10.2 (8.7–11.7)	11.4 (7.7–15.1)	5.7	13.4	8.6	0.13

Categorical variables were analyzed using counts and percentages. Continuous variables were summarized using medians and corresponding 95% CIs. SNA—angle between sella, nasion, and point A; SNB—angle between sella, nasion, and point B; ANB—angle formed by point A, nasion, and point B; WITS appraisal—distance between perpendicular lines drawn from point A (maxilla) and point B (mandible) to the occlusal plane; SN/NL—angle between sella–nasion line and nasal line (spina nasalis anterior–pterygomaxillare). * Paired samples *t*-tests were performed with a significance level of <0.05.

## Data Availability

All information and data are presented within this manuscript.
